# Polymeric Nanomaterials for Efficient Delivery of Antimicrobial Agents

**DOI:** 10.3390/pharmaceutics13122108

**Published:** 2021-12-07

**Authors:** Yin Wang, Hui Sun

**Affiliations:** 1 School of Public Health and Management, Ningxia Medical University, Yinchuan 750004, China; wy9522@126.com; 2 State Key Laboratory of High-Efficiency Coal Utilization and Green Chemical Engineering, Ningxia University, Yinchuan 750021, China

**Keywords:** antimicrobial agent, polymeric nanomaterial, self-assembly, antimicrobial peptide, silver nanoparticle, anti-biofilm, wound healing, multidrug resistance

## Abstract

Bacterial infections have threatened the lives of human beings for thousands of years either as major diseases or complications. The elimination of bacterial infections has always occupied a pivotal position in our history. For a long period of time, people were devoted to finding natural antimicrobial agents such as antimicrobial peptides (AMPs), antibiotics and silver ions or synthetic active antimicrobial substances including antimicrobial peptoids, metal oxides and polymers to combat bacterial infections. However, with the emergence of multidrug resistance (MDR), bacterial infection has become one of the most urgent problems worldwide. The efficient delivery of antimicrobial agents to the site of infection precisely is a promising strategy for reducing bacterial resistance. Polymeric nanomaterials have been widely studied as carriers for constructing antimicrobial agent delivery systems and have shown advantages including high biocompatibility, sustained release, targeting and improved bioavailability. In this review, we will highlight recent advances in highly efficient delivery of antimicrobial agents by polymeric nanomaterials such as micelles, vesicles, dendrimers, nanogels, nanofibers and so forth. The biomedical applications of polymeric nanomaterial-based delivery systems in combating MDR bacteria, anti-biofilms, wound healing, tissue engineering and anticancer are demonstrated. Moreover, conclusions and future perspectives are also proposed.

## 1. Introduction

Infectious diseases induced by bacteria, virus and fungi have been considered as one of the biggest enemies that threatened the lives of human beings for a long time [1]. Since the discovery of penicillin in 1928, antibiotics have played an unprecedented role in saving lives of human beings and caused revolutionary changes in medicine. However, with overuse and improper use of antibiotics, the emergence of bacterial drug resistance is becoming a severe problem. In particular, combating MDR bacteria such as methicillin-resistant *Staphylococcus aureus* (*S. aureus*) (MRSA) has drawn wide attention and efforts [2,3]. Non-antibiotic antimicrobial agents such as AMPs [4,5,6], silver nanoparticles (AgNPs) [7,8,9], metal oxides [10,11,12], antimicrobial peptoids [13,14] and polymers [15,16,17,18] are alternatives for treating infectious diseases that kill bacteria in a physical manner and avoid the generation of drug resistance. For instance, cationic compounds including AMPs, antimicrobial peptoids and polymers, as well as their corresponding nanostructures, strongly interacted with the negatively charged cell membrane of bacteria, resulting in the disruption of the cell membrane and outflow of the content of bacteria [19,20]. Metal (oxide) nanoparticles such as widely studied AgNPs kill bacteria via heavy metal ions induced by the denaturation of proteins or genetic materials, while ZnO and TiO_2_ nanoparticles eliminate bacteria by reacting with reactive oxygen species (ROS) generated from photocatalytic process [12,21]. Moreover, emerging antimicrobial agents including gases, photothermal sensitizers and carbon materials were also developed to combat bacterial infections [17,22,23,24].

The efficient delivery of antimicrobial agents to the target preventing the defense system of bacteria including efflux pump, degrading enzymes and resistance genes is critical for reducing the emergence of drug resistance [25,26,27]. Polymeric nanomaterials are promising vehicles for the efficient delivery of antimicrobial agents due to their tailorable chemical compositions, microstructures and biological properties for a wide range of biomedical applications [28,29,30]. For instance, low dimensional nanostructures including dendrimers [31,32], polymeric nanoparticles [33,34], micelles [35,36], vesicles [37,38] and nanogels [39,40] have shown superiorities in the delivery of antimicrobial agents to the areas of infections and on-demand release. Polymeric nanofibers and hydrogels are beneficial for the long-term release of antimicrobial agents and wound coverage [41,42]. Very recently, metal organic frameworks (MOFs) have attracted attention as emerging carriers for the efficient delivery of metal ions, metal nanoparticles, antibiotics and enzymes due to their highly porous structures [43,44,45,46]. There are several advantages of using polymeric nanomaterials as carriers to accomplish the on-demand delivery of antimicrobial agents: (i) reduced dosage and drug resistance; (ii) increased in vivo circulation stability; (iii) enhanced penetration ability; (iv) prolonged antimicrobial performance; and (v) improved bioavailability. Therefore, apart from the wide attentional broad spectrum antimicrobial properties, biomedical applications including combating MDR bacteria, anti-biofilm, anticancer, wound healing and tissue engineering based on the polymeric antimicrobial agent delivery systems have been rapidly developed [47,48,49,50].

**Figure 1:**
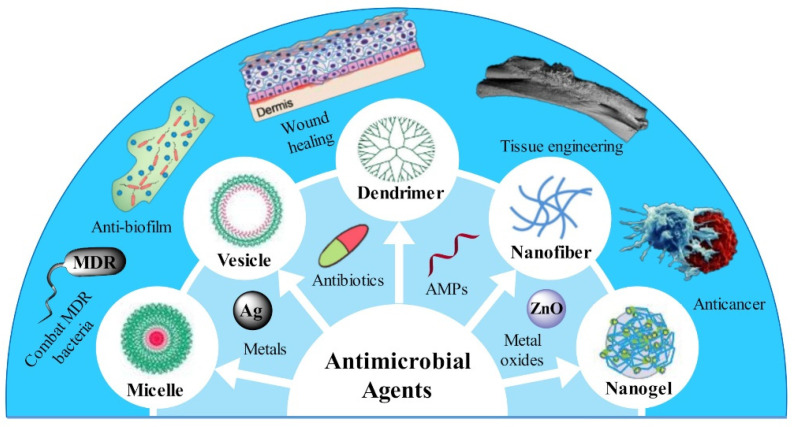
Schematic illustration of polymeric nanomaterials for efficient delivery of antimicrobial agents and their biomedical applications.

In this review, we aim to present the state of the art of polymeric nanomaterials as carriers for the efficient delivery of antimicrobial agents from the following aspects: (1) classification of polymeric nanoparticles based on their nanostructures; (2) the structural features and corresponding advantages in delivery of antimicrobial agents; and (3) biomedical applications benefiting from the constructed delivery systems, as illustrated in Figure 1.

## 2. Efficient Delivery of Antimicrobial Agents by Diverse Polymeric Nanostructures

### 2.1. Self-Assembled Polymeric Nanoparticles

Polymer self-assembly has been recognized as one of the most versatile strategies for preparing soft nanomaterials with various morphologies and functionalities from small building blocks [51,52,53,54,55,56]. Typically, polymer micelles and vesicles are the most easily obtained and widely studied nano-objects due to their well-organized structures [57,58,59,60]. Polymer micelles are formed by the regular arrangement of building blocks with hydrophobic components forming the cores and hydrophilic polymer chains covering the surface. Moreover, the hydrophobic cores facilitated the efficient encapsulation of hydrophobic drugs [61,62], while polymer vesicles are hollow bilayer nanostructures with hydrophobic membranes, hydrophilic coronas and interior cavities, endowing them with superiorities in loading and delivering hydrophobic, hydrophilic and large-sized cargoes [63,64,65,66]. The design of polymer vesicles for meeting the requirements of various applications mainly focuses on the chemical composition and structure of coronas and membranes, such as permeability and homogeneity of the membrane, symmetricity of the corona and so forth [67,68,69]. Despite the wide applications of polymer micelles and vesicles in cancer therapy, gene delivery and cell imaging, they also exhibit considerable potentials in the efficient delivery of antimicrobial agents [36,70,71].

**Figure 2:**
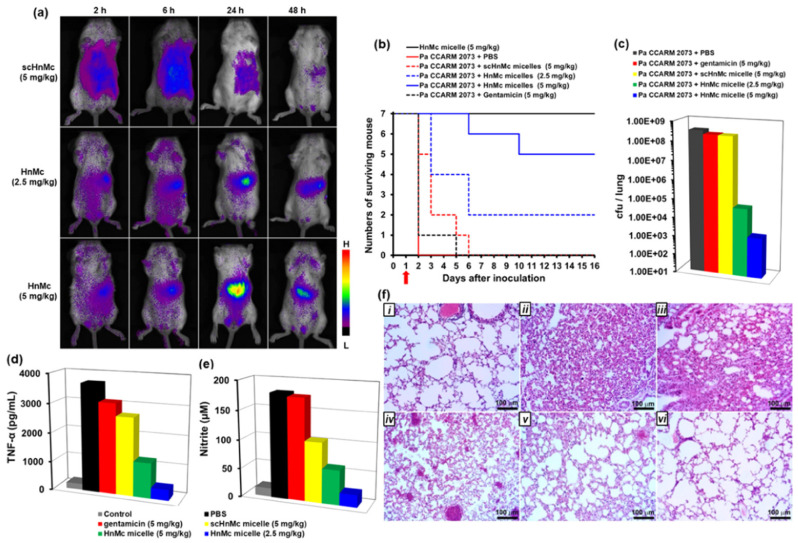
Targeted antibacterial activity of HnMc micelles in the mouse model of drug resistant *P. aeruginosa* lung infection. (**a**) Fluorescent imaging of infected mice after intravenous injection of fluorescent IR820-loaded HnMc micelles. (**b**) Survival rate of infected mice after administration of HnMc micelles. (**c**) Number of remaining cells in the infected lungs after administration of HnMc micelles. Inhibitory effects of HnMc micelles on the expression of TNF-α (**d**) and nitric oxide (**e**) in the blood of infected mice. (**f**) H&E-stained lung tissues. (**i**): control; (**ii**): *P. aeruginosa* + PBS; (**iii**): *P. aeruginosa* + gentamicin (5 mg kg^−1^); (**iv**): *P. aeruginosa* + scrambled HnMc micelle (5 mg kg^−1^); (**v**): *P.* aeruginosa + HnMc micelle (2.5 mg kg^−1^); and (**vi**): *P. aeruginosa* + HnMc micelle (5 mg kg^−1^) (reproduced with permission from Park et al. [79], ACS Applied Materials & Interfaces; published by American Chemical Society, 2020).

Polymer micelles, core-shell nanostructures that usually self-assembled from amphiphilic block copolymers, are regarded as one of the most extensively studied nanostructures for antimicrobial agent delivery [35,72]. Typically, hydrophobic antibiotics, AMPs and AgNPs can be loaded in the hydrophobic core, and amphiphilic antimicrobial molecules are usually decorated on the surface of polymer micelles by covalent bonding or electrostatic interaction [73,74,75,76]. For instance, a poor water-soluble anti-fungi agent, amphotericin B, could be encapsulated in the core of micelles and showed ultra-long sustained release for 150 h, resulting in reduced hemotoxicity and comparable anti-fungi activity compared with free amphotericin B [77]. Xiong and coworkers [78] functionalized terpyridine on the surface of polymer micelles; after chelating with Fe(II), the micelles displayed excellent biofilm inhibition activity up to 99.9% at a concentration of 128 μM. Recently, Lee et al. [79] prepared AMP-covered micelles by the co-assembly of chimeric antimicrobial lipopeptide and a biodegradable amphiphilic polymer (poly-(lactic-*co*-glycolic acid)-*b*-poly(ethylene glycol), (PLGA-*b*-PEG)). The chimeric peptide HnMc and PEG formed the shell of the micelles in which PEG protected HnMc from proteolytic degradation. Moreover, HnMc on the surface could help micelles in preferentially binding and killing bacteria. Due to the synergy between HnMc and PEG, the micelles targeted a wide range of bacteria preferentially including *Escherichia coli* (*E. coli*), *Listeria monocytogenes*, *Pseudomonas aeruginosa* (*P. aeruginosa*) and *S. aureus* instead of mammalian cells. Moreover, in vivo experiments also demonstrated superior anti-inflammatory effects of the micelles in a mouse model of drug-resistant *P. aeruginosa* lung infection with highly targeted abilities, as shown in Figure 2.

**Figure 3:**
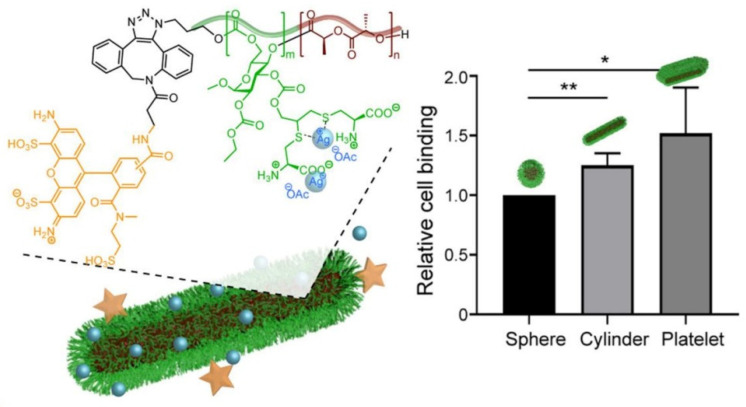
Polymer micelles, cylinders and nanoplates derived from the CDSA of PDGC-*b*-PLLA and their antimicrobial activity (* *p* < 0.05 and ** *p* < 0.01 by *t* test) (reproduced with permission from Song et al. [80], Nano Letters; published by American Chemical Society, 2021).

Very recently, Wooley and coworkers [80] fabricated spherical micelles, cylinders and nanoplates derived from the crystallization-driven self-assembly (CDSA) of an amphiphilic block copolymer composed of zwitterionic poly(ᴅ-glucose carbonate) and semicrystalline poly(ʟ-lactide) segments (PDGC-*b*-PLLA). As illustrated in Figure 3, fluorescent molecule and cysteine were modified on the polymer in order to afford tracing ability and to chelate with silver ions, respectively. The morphology of the nanostructures could be well controlled by the hydrophilic-to-hydrophobic ratios, which exhibited negligible cytotoxicity, immunotoxicity and cytokine adsorption. However, the nanostructures offered substantial silver ion loading capacity, extended release and in vitro antimicrobial activity. Compared with spherical micelles, the cylinders and nanoplates exhibited enhanced association with uroepithelial cells due to their high aspect ratio, resulting in improved inhibition of the growth of *E. coli* in recurrent urinary tract infections.

Compared with polymer micelles, polymer vesicles are closed hollow spheres with more complicated structures usually acting as simple mimics of biological cells [81]. There are three compartmentalized regions that should be considered for realizing different functions, namely the inner hydrophilic cavity, hydrophobic membrane and hydrophilic corona in contact with external environments [82]. Therefore, both hydrophilic and hydrophobic compounds and even nanoparticles could be encapsulated in the interior cavity or membrane of vesicles, respectively. Moreover, hydrophilic molecules could also be linked onto the coronas of polymer vesicles by covalent bonding. Considering the structural feasibility of polymer vesicles, a large variety of antimicrobial agents could be loaded and delivered to combat bacteria with high loading efficiency, controlled release manner, targeting capability and improved bioavailability [83,84]. For example, Du and coworkers [35] deposited ultrafine AgNPs with a diameter of 1.9 ± 0.4 nm on the membrane of polymer vesicles by in situ reduction of silver ions to inhibit the growth of Gram-negative and Gram-positive bacteria. Battaglia et al. [71] reported the intracellular delivery of metronidazole or doxycycline to *P. gingivalis*-infected oral epithelial cells by polymer vesicles, which were disassembled in early endosomes due to the acidic condition, resulting in the release of loaded cargoes.

**Figure 4:**
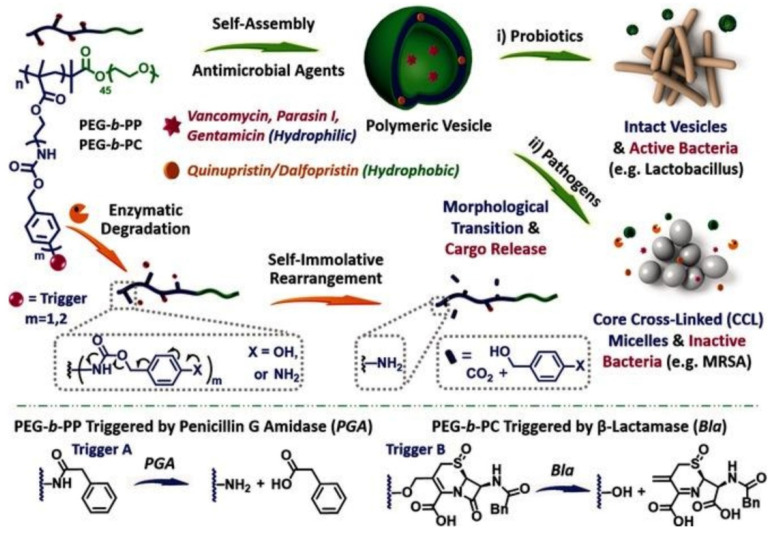
Enzyme-responsive polymer vesicles for bacterial strain-selective delivery of antimicrobials (reproduced with permission from Li et al. [85], Angewandte Chemie International Edition; published by Wiley, 2015).

Recently, Liu and coworkers [85] designed enzyme-responsive polymer vesicles for bacterial strain-selective delivery of antimicrobials, as shown in Figure 4. Both hydrophilic and hydrophobic antimicrobials including vancomycin, gentamicin, quinupristin and dalfopristin could be encapsulated either in the interior cavity or membrane of the polymer vesicles with high efficiency. The PEG chains covered on the surface of the vesicle could reduce cytotoxicity and improve biocompatibility, while the self-immolative side chains could be degraded by penicillin Gamidase (PGA) and *β*-lactamase (Bla), which are overexpressed by drug resistant bacterial strains. Without the trigger by PGA and Bla, the encapsulated antimicrobials were well protected by vesicles. Upon being exposed to drug-resistant bacteria, the membrane of the vesicle was degraded, resulting in the sustained release of antimicrobials, as well as the elimination of bacteria. Considering that Bla is the main cause of bacterial resistance to *β*-lactam antibiotic drugs that are secreted by MRSA, selective antimicrobial activity of the antimicrobials-loaded vesicles was achieved.

Loading bioactive enzymes by polymer vesicles to generate antimicrobial active species triggered by external stimuli is another effective method for combating bacteria. For example, Blackman et al. [86] prepared glucose oxidase-loaded semipermeable polymer vesicles by polymerization-induced self-assembly inspired by honey. Hydrogen peroxide, an effective antimicrobial agent, could be generated in response to glucose to switch on antimicrobial activity of the vesicles. In the absence of glucose, the vesicles were completely nontoxic to bacteria, while the vesicles showed seven-log reduction in bacterial growth at high glucose concentrations against a range of Gram-negative and Gram-positive bacterial pathogens including *S. aureus*, *S. epidermidis*, *E. coli* and *Klebsiella pneumoniae* (*K. pneumonia*), even the MRSA clinical isolate. More importantly, the toxicity of the vesicle toward human fibroblasts at different dosage and glucose concentrations was also evaluated, demonstrating that the optimal concentration of the vesicle was 0.69 mg mL^−1^ at physiological blood glucose level to effectively eliminate bacteria while preserving good compatibility to mammalian cells.

### 2.2. Dendrimers

Dendrimers are highly branched, globular macromolecules with many arms emanating from a central core, which have shown unique structural properties such as high degree of branching, multivalency, globular architecture and well-defined molecular weight, rendering them promising scaffolds for drug delivery [87,88]. Many commercial drugs with anticancer and antimicrobial activity have been successfully loaded within dendrimers including poly(amidoamine) (PAMAM), poly(propylene imine) (PPI) and poly(etherhydroxylamine) (PEHAM), either via physical interactions or by chemical bonding to improve their water solubility [89]. Dendrimers themselves could be used as effective antimicrobial agents [90]. For instance, those with positively charged surfaces usually have strong interaction with negatively charged bacterial cell membranes, while those with metal cores can release active antimicrobial agents such as metal ions and ROS, resulting in the death of bacteria [91].

Moreover, antimicrobial agents including antibiotics, AMPs, AgNPs and metal oxide nanoparticles could be also effectively loaded by dendrimers [89,92]. For example, Tang et al. [93] prepared silver-dendrimer nanocomposites by loading AgNPs in low generation poly(amido amine) dendrimers. The AgNPs were formed by an in situ reduction of silver ions enriched by the amine groups of dendrimers. The factors that influenced the size of AgNPs were discussed, and the average diameter of the AgNPs could be controlled from 7.6 to 16.2 nm. The synthesized silver-dendrimer nanocomposite was used as antimicrobial agent in the fabrication of cotton fabrics, which exhibited excellent antimicrobial activity against both of *E. coli* and *S. aureus*. Recently, Huang and coworkers [94] reported PLGA nanoparticles and PAMAM dendrimers in order to effectively encapsulate and deliver platensimycin, a potent inhibitor for the synthesis of bacterial fatty acid, respectively, to combat MDR bacteria. Benefiting from the improved pharmacokinetics, both the platensimycin-loaded PLGA nanoparticles and PAMAM dendrimers showed enhanced antimicrobial activity and reduced cytotoxicity compared with free platensimycin, resulting in an efficient inhibition of *S. aureus* biofilm formation and the full survival of MRSA-infected mice.

**Figure 5:**
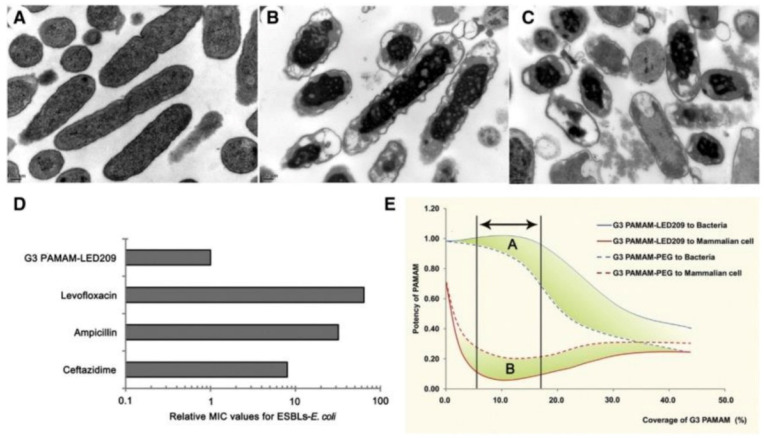
The morphology of EHEC was investigated by transmission electron microscopy (TEM) after different treatments for 300 min. (**A**) Control; (**B**) 75 μg mL^−1^ of G3 PAMAM; and (**C**) 150 μg mL^−1^ of G3 PAMAM-LED209. (**D**) Induction of resistance to G3 PAMAM-LED209. (**E**) The influence of the conjugated ligand and degree of modification on cytotoxicity and antibacterial activity of terminally modified G3 PAMAM dendrimer. Area A and B showed increased antibacterial activity and reduced cytotoxicity with respect to G3 PAMAM-LED209 and G3 PAMAM-PEG, respectively (reproduced with permission from Xue et al. [98], Nanomedicine: Nanotechnology, Biology and Medicine; published by Elsevier, 2015).

Dendrimers are ideal platforms for compacting and delivering deoxyribonucleic acids (DNAs) and ribonucleic acids (RNAs) for gene therapy due to their hyperbranched structure and strong positive charges, especially PAMAM [95,96]. Recently, antisense therapy strategy has been developed to treat bacterial infections facilitated by the dendrimers-based antisense delivery system [97]. For example, the G3 PAMAM dendrimer has good antimicrobial activity, as shown in Figure 5. However, the cytotoxicity of the G3 PAMAM dendrimer toward mammalian cells is also high. Luo et al. [98] conjugated LED209, a specific inhibitor of quorum sensor QseC of Gram-negative bacteria, onto the surface of G3 PAMAM to generate PAMAM-LED209 in order to reduce cytotoxicity to mammalian cells while retaining the excellent antibacterial activity of the G3 PAMAM dendrimer. In addition, PAMAM-LED209 also inhibited the virulence gene expression of Gram-negative bacteria and prevented the generation of drug resistance. As shown in Figure 5, compared with the control group (Figure 5A), *entero-hemorrhagic E. coli* (*EHEC*) were severely damaged after being treated with G3 PAMAM and G3 PAMAM-LED209 for 300 min (Figure 5B,C), demonstrating that G3 PAMAM-LED209 retained strong antibacterial activity toward resistant Gram-negative bacteria after functionalization of LED209. The induction of the resistance of G3 PAMAM-LED209 was also evaluated after 15 reproductions of bacteria, as illustrated in Figure 5D. The minimal inhibition concentration (MIC) of G3 PAMAM-LED209 barely changed, while the MIC values of classical antimicrobials, including ceftazidime, ampicillin and levofloxacin, increased by 8-fold to 64-fold. The cytotoxicity and antibacterial activity of terminally modified PAMAM are related to the conjugated ligand and degree of modification, as shown in Figure 5E. With an increase in modification ratio, the cytotoxicity of G3 PAMAM-PEG and G3 PAMAM-LED209 decreased dramatically to being almost nontoxic and then increased, while the antimicrobial activity of the G3 PAMAM-PEG and G3 PAMAM-LED209 decreased with an increase in modification ratio due to the shielding of positive charges. Therefore, there is an optimal modification ratio range for balancing cytotoxicity and antimicrobial activity, as pointed out by the arrow in Figure 5E. Moreover, the antibacterial potency of G3 PAMAM-LED209 is also higher than that of G3 PAMAM-PEG, which is indicated by area A and B in Figure 5E, demonstrating better biocompatibility and higher antibacterial potency than compared to G3 PAMAM-PEG.

### 2.3. Polymer Nanofibers

Polymer nanofibers are one dimensional nanostructures with large aspect ratio and high surface area and have shown significant potential for delivering antimicrobial agents locally into an infected area, especially in wound healing [42,99]. Typically, there are several methods for preparing nanofibers including self-assembly [100], template synthesis [101], phase separation [102] and electrospinning [103], among which electrospinning is a superior technique for preparing nanofibers with desired chemical compositions and diameters due to its simplicity and versatility [104,105,106]. Antimicrobial agents including antibiotics, AMPs, AgNPs and metal oxide nanoparticles could be incorporated into nanofibers by mixing with polymer precursors followed by electrospinning or attaching onto the surface of the nanofibers by noncovalent interactions or chemical bonds [107]. For instance, Schiffman et al. [108] immobilized zeolites nanoparticles with high silver ion change capability onto the surface of chitosan nanofibers. After ion exchange, silver ions were loaded in the zeolites to function as molecular delivery vehicles, and their ion release profiles and ability to inhibit *E. coli* were evaluated as a function of time. Interestingly, the zeolites immobilized on the nanofibers showed significantly enhanced antibacterial activity 11-times greater than that of the pure zeolites due to high porosity and hydrophilicity of the nanofibers.

**Figure 6:**
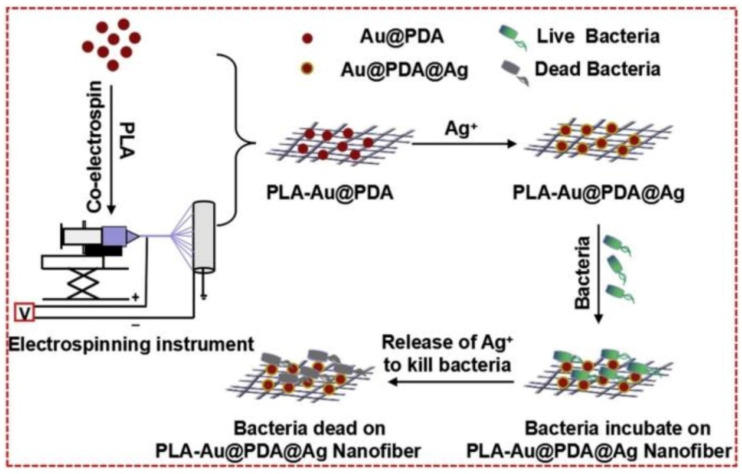
Schematic diagram illustrating the preparation of PLA-Au@PDA@Ag nanofibers and their antibacterial capacity (reproduced with permission from Zhang et al. [109], Colloids and Surfaces B: Biointerfaces; published by Elsevier, 2019).

Recently, Tu and coworkers [109] reported the in situ deposition of AgNPs on gold/polydopamine core-shell nanoparticles encapsulated by poly(lactic acid) (PLA) nanofibers (PLA-Au@PDA@Ag), which could be applied to biological coatings for bacteriostatic functionality. The schematic illustration of the preparation and antimicrobial capability of the PLA-Au@PDA@Ag is presented in Figure 6. Chloroauric acid was reduced by ascorbic acid to afford gold nanoparticles. Following the polymerization of dopamine on the surface, Au@PDA core-shell nanoparticles formed, which were then mixed with PLA solution to produce PLA-Au@PDA hybrid nanofibers by electrospinning. Later, PLA-Au@PDA hybrid nanofibers were immersed in silver nitrate solution for in situ reduction of adsorbed silver ions into AgNPs to yield PLA-Au@PDA@Ag nanofibers. The hydrophilicity of the PLA-Au@PDA@Ag nanofibers significantly improved compared to that of PLA nanofibers, resulting in the promoted release of silver ions. Benefiting from the synergy between AuNPs, PDA and AgNPs, including AuNPs providing effective contact with microorganisms, PDA as binder was used to immobilize AgNPs and facilitated the release of silver ions; the PLA-Au@PDA@Ag nanofibers showed significant antibacterial ability against both of *E. coli* and *S. aureus*.

Due to their large exposed surface area and nanoporosity, polymer nanofiber meshes have shown distinct advantages in wound healing compared with hydrogels, films and foams [110]. The extracellular matrix (ECM) mimicking the structure of nanofibers facilitated the interaction with cells in the wound bed. Moreover, small molecules such as water, oxygen, nutrients and metabolic wastes could be efficiently exchanged due to the highly porous structure of nanofibers [111]. In order to promote the healing rate and elimination of bacteria, functional agents including enzymes, drugs and antimicrobial agents have been incorporated in polymer nanofibers. Rath et al. [112] loaded ZnO nanoparticles and cefazolin in the gelatin nanofibers to accelerate wound healing and prevented infection concurrently. Cefazolin was used to inhibit bacterial reproduction, while zinc cations could be released from ZnO nanoparticles to raise re-epithelialization, reduce inflammation and inhibit bacterial growth. Moreover, ROS was also produced by ZnO nanoparticles, thereby optimizing cell adhesion, proliferation and migration via growth factor mediated pathways, promoting the regeneration of the ECM.

### 2.4. Polymer Nanogels

Polymer nanogels are a class of nanoparticles composed of nanosized physically or chemically cross-linked hydrophilic or amphiphilic polymer networks [113]. They are of wide interest in various fields including drug delivery due to their flexible nanosize, good stability and high loading capacity, etc. [114]. As their analogues, polymer hydrogels have been widely used in antimicrobial applications due to their high water content, three-dimensional structure and stimuli-responsive sol-gel transition behavior [115]. There are several reviews summarizing the recent advances of antimicrobial polymer hydrogels [116,117,118]. Therefore, we will not discuss this part and focus on the nanogels as carriers for antimicrobial agent delivery in this section.

The stimuli-responsive swelling and collapsing of nanogels triggered by external stimuli including pH, temperature, enzymes or ionic strength render them ideal candidates in on-demand delivery and release of antimicrobial agents [119]. For instance, AMPs could be encapsulated in nanogels with high loading content via strong electrostatic interaction with negatively charged polymer chains, and they can be released when triggered by salt ions in physiological conditions [120,121]. El-Feky et al. [122] loaded silver sulfadiazine in alginate coated chitosan nanogels to heal burn wounds, and the nanogels showed a release profile of an initial burst followed by a slow and continuous release, resulting in excellent in vivo therapeutic efficacy.

**Figure 7:**
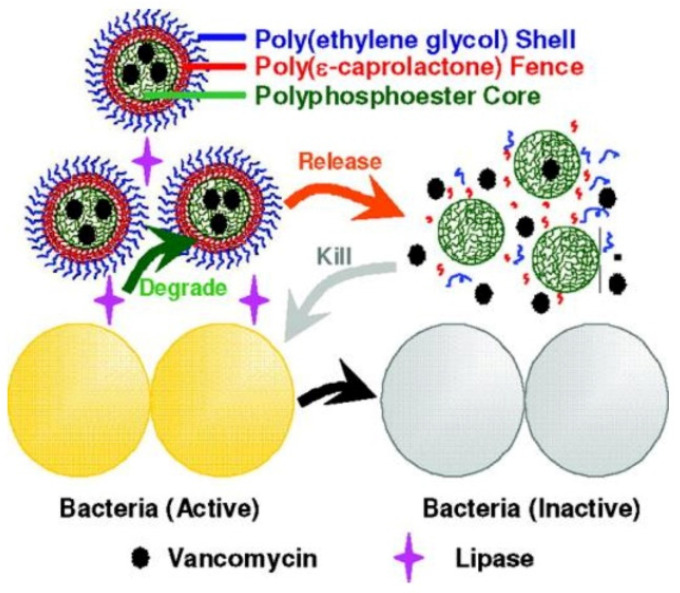
Schematic illustration of the on-demand delivery of vancomycin triggered by bacterial lipase to treat the bacterial infections using TLNs, which contains a bacterial lipase-sensitive PCL interlayer between the cross-linked polyphosphoester core and the shell of the PEG (reproduced with permission from Xiong et al. [127], Journal of the American Chemical Society; published by American Chemical Society, 2012).

In addition, loading and delivery of antimicrobials including berberine, cyclodextrin, tetracycline hydrochloride and lincomycin hydrochloride by nanogels to combat bacteria and MDR bacteria were widely studied by Paunov, Schaefer and so forth [123,124,125,126]. Wang and coworkers [127] designed a lipase-sensitive polymeric triple-layered nanogel (TLN) formed by a cross-linked polyphosphoester core, poly(*ε*-caprolactone) (PCL) fence and PEG shell to encapsulate and deliver vancomycin, as illustrated in Figure 7. In aqueous solutions, hydrophobic PCL segments collapsed and covered the core to form a densely packed molecular fence to prevent the leakage of vancomycin. Once TLN was exposed to lipase secreting bacteria, the PCL chains were degraded to trigger the release of vancomycin, resulting in the inhibition of bacterial growth. They found that all encapsulated vancomycins were released within 24 h in the presence of *S. aureus*. Moreover, lipase secreting bacteria inside the cells could also be inhibited by TLN, demonstrating the versatility of the strategy of lipase-induced on-demand delivery and release of antimicrobials.

**Figure 8:**
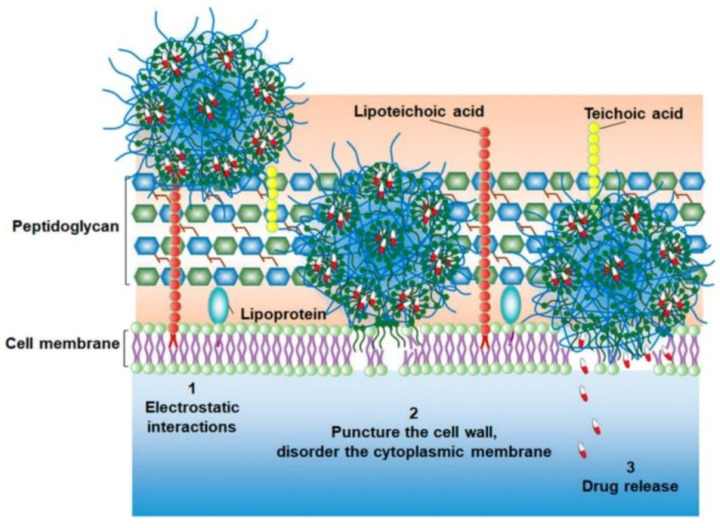
Schematic illustration of the bactericidal mechanism of Triclosan-loaded nanogels (adapted from Zu et al. [129], ACS Applied Polymer Materials; published by American Chemical Society, 2020).

Recently, Knowles et al. [128] synthesized hybrid organic/inorganic AgNPs loaded nanofibrillar silk microgels to effectively eradicate bacteria by a two-step mechanism including bacterial adherence and consequent eradication. Compared with conventional AgNPs and silver ions, the hemolysis and cytotoxicity of hybrid microgels toward mammalian cell lines were significantly reduced due to the protection of the silk matrix. van Rijn and coworkers [129] prepared injectable nanogels loaded with hydrophobic triclosan in hydrophobic domains inside the nanogel networks through intraparticle self-assembly of aliphatic chains, which enhanced antimicrobial efficiency of triclosan up to 1000 times. As shown in Figure 8, a three-stage antimicrobial mechanism of the nanogels was proposed. Firstly, the nanogels attached onto the surfaces of the bacteria via electrostatic interaction to disturb the balance of charge density of the cell membranes. Secondly, bacterial cell membranes were destroyed by the insertion of hydrophobic aliphatic chains. Thirdly, loaded triclosan was released from the hydrophobic domains inside the nanogels and injected into the bacterial cell membranes, resulting in the death of bacteria. This approach dramatically increases the effective concentration of triclosan inside the bacteria. Moreover, both the MIC and minimal bactericidal concentration (MBC) against Gram-positive *S. aureus* and *S. epidermidis* decreased by three orders of magnitude compared with free triclosan, resulting in a decrease in the dosage of triclosan and reduction in drug resistance.

### 2.5. Hybrid Delivery Systems

Incorporating polymer nanoparticles including dendrimers, micelles and vesicles with high dimensional polymeric nanomaterials such as nanofibers, hydrogels and coatings as hybrid delivery systems could combine the advantages of both and achieve the hierarchical release of antimicrobial agents [130,131,132,133]. For example, Zhang and coworkers [130] developed a bioadhesive nanoparticle-hydrogel hybrid in order to enhance localized antimicrobial drug delivery. The antimicrobials ciprofloxacin was loaded in polymer nanoparticles that were embedded in hydrogels adhering to biological surfaces. Hydrogel network properties could be tailored independently for adhesion, which maintained controlled and prolonged ciprofloxacin release profiles from nanoparticles. Imae et al. [131] immobilized AgNPs-loaded amine-terminated fourth generation poly(amido amine) dendrimers onto the viscose rayon cellulose fibers, which exhibited excellent biocidal activity against *E. coli* with low weight percentage of silver of 0.2%. Du and coworkers [132] embedded penicillin encapsulated polypeptide polymersomes in the hydrogels to achieve quick and long-term antibacterial capability in which penicillin could be released from the hydrogel networks for quick bacteria elimination while the intrinsic antibacterial property of the polymersomes ensured long-term antibacterial activity. However, despite the advantages of hybrid delivery systems, the development of incorporation of different polymeric nanostructures as hybrid delivery platforms is still in its infancy, which may bring new opportunities in efficient loading and delivery of antimicrobial agents.

## 3. Biomedical Applications of Polymeric Nanomaterials Based Antimicrobial Agent Delivery Systems

### 3.1. Combating MDR Bacteria

The generation of drug resistance of pathogens is typically caused by the accumulation of drug resistant genes through mutation with the long-term use, especially overuse and improper use of antibiotics [25]. Therefore, the exploration of highly efficient delivery system to reduce dosage and improve bioavailability of antibiotics, as well as the delivery of non-antibiotic antimicrobial agents including AMPs, AgNPs, metal oxides, gases and so forth, is a promising strategy for reducing drug resistance [134,135]. Polymeric nanomaterial-based antimicrobial agent delivery systems have widely been used in combating MDR bacteria [136,137]. For instance, Liu et al. [138] conjugated quercetin and acetylcholine on the surface of selenium nanoparticles to combat MDR bacteria, which could effectively eliminate MRSA by destroying the membrane due to the synergy between quercetin, acetylcholine and selenium nanoparticles. Cationic polymeric star-shaped nanoparticles or dendrimers have also shown excellent antimicrobial activity against MDR bacteria even without loading antimicrobial agents [139,140,141], demonstrating the great potentials of polymeric nanomaterials in combating MDR bacteria.

**Figure 9:**
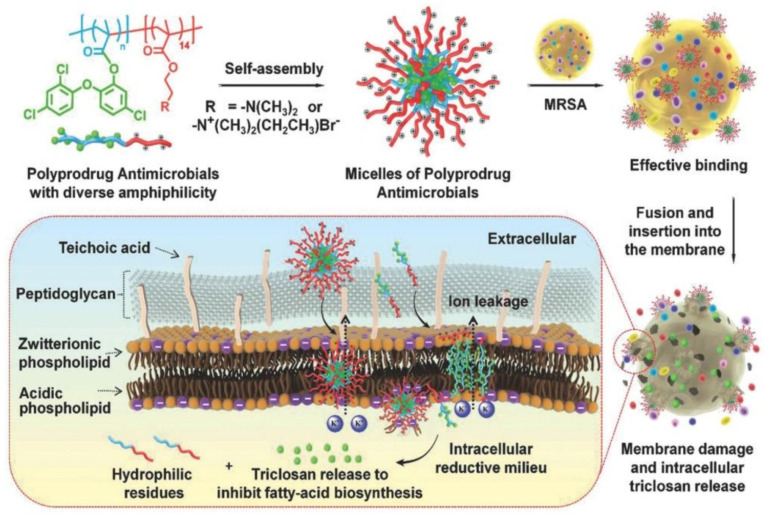
The polyprodrug antimicrobials with optimized hydrophilic–hydrophobic balance for efficiently eradicating MRSA with remarkable membrane damage and concurrent drug release profiles (reproduced with permission from Cao et al. [142], Small; published by Wiley, 2018).

Hu et al. [142] prepared polyprodrug antimicrobials to combat MRSA by membrane damage and concurrent drug release, as shown in Figure 9. Triclosan was covalently linked with acrylic acid to produce a triclosan prodrug monomer (TMA). Then, TMA was copolymerized with quaternized *N*,*N*-dimethylaminoethyl methacrylate (QDMA), affording PQDMA-*b*-PTMA, which could self-assemble into prodrug micelles with positively charged surfaces. The hydrophilic–hydrophobic balance of the prodrug micelles was optimized to enhance interaction with bacterial cell membranes, resulting in improved antimicrobial activity. They proposed that the antimicrobial mechanism was as follows: (1) the prodrug micelles attached onto the surface of MRSA due to strong electrostatic interaction; (2) the prodrug micelles fused with and inserted into the cell membrane of MRSA; (3) the cell membrane of MRSA was damaged due to charge disorder, and prodrug micelles were encapsulated into the cell; (4) prodrug micelles were disassembled, and the linkage between triclosan and acrylic acid was broken due to the reductive milieu environment, resulting in the in situ release of triclosan and death of MRSA. It was noteworthy that no detectable resistance was observed due to the synergistic antibacterial mechanism, and prodrug micelles exhibited remarkable bacterial inhibition and low hemolysis toward red blood cells compared with commercial triclosan and vancomycin.

**Figure 10:**
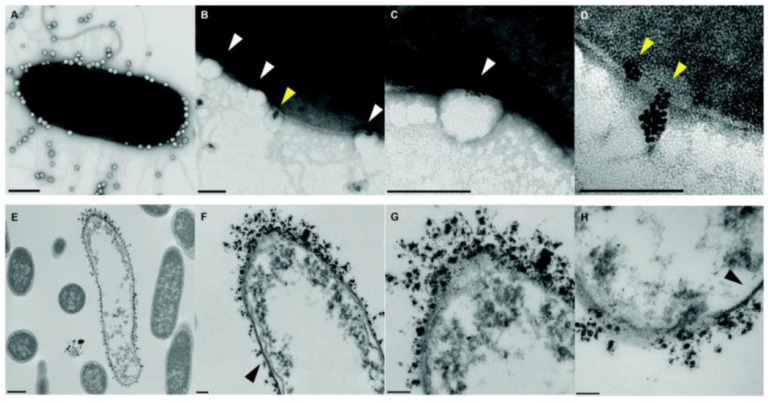
TEM images of bacteria-polymer vesicles interactions. Scale bars are 100 nm for (**A**–**D**,**F**–**H**) and 500 nm for (**E**), respectively. White arrows pointed out the indentation of bacterial cell membrane in regions of AgNPs loaded polymer vesicles; yellow arrows implied the polarization indicative of hydrophobic interactions of AgNPs inside the vesicles; black arrows revealed that the regions of the outer membrane with little AgNPs loaded polymer vesicles contact appeared morphologically normal (reproduced with permission from Geilich et al. [145], Nanoscale; published by Royal Society of Chemistry, 2015).

The combination of different classes of antimicrobial agents such as antimicrobials and AgNPs could afford synergistic effects, resulting in the efficient inhibition of MDR bacteria that is far better than its individual components [143,144]. Webster and coworkers [145] prepared polymer vesicles to co-deliver ampicillin and AgNPs simultaneously in the hydrophilic cavity and hydrophobic membrane, respectively. The AgNPs-embedded polymersomes exhibited potent antibacterial activity against *E. coli* transformed with a gene for ampicillin resistance in a dose-dependent fashion, while the free ampicillin, AgNPs decorated polymersomes without ampicillin and ampicillin loaded polymersomes without AgNPs had no effect on bacterial growth. TEM images in Figure 10 revealed that the interactions between vesicles, AgNPs and bacterial cells might result in the deformation and disruption of bacterial envelopes and consequently result in the death of bacteria. Later, the same group [146] functionalized proline-rich AMP PR-39 on the corona of polymer vesicles with AgNPs embedded in the membrane to combat MRSA with a AMP/AgNPs ratio-dependent behavior. A ratio of AgNPs-to-AMP of 1:5.8 corresponding to 11.6 μg mL^−1^ of AgNPs and 14.3 × 10^−6^ M of AMP exhibited the best MRSA inhibition activity, demonstrating the potentials of binary or ternary antimicrobial agent co-delivery systems in combating MDR bacteria.

### 3.2. Anti-Biofilm

Biofilms are matrix-enclosed communities of bacteria that show increased drug resistance and capability to evade the immune system [47]. It has been widely recognized that bacteria exist in the form of biofilms in many instances, which is hard to eliminate due to the protection of extracellular polymeric substances (EPS), a complex matrix composed of proteins, nucleic acids, phospholipids, polysaccharides, blood components and humic substances produced by bacteria [147]. Therefore, it is difficult for antimicrobials to penetrate the EPS to kill bacteria, resulting in the occurrence of drug resistance. The efficient delivery of antimicrobial agents by polymeric nanomaterials is considered a promising strategy for penetrating the biofilm and delivering antimicrobial agents to the deep end of the matrix to kill pathogens [148,149,150]. For example, Deoxyribonuclease I functionalized ciprofloxacin-loaded PLGA nanoparticles were prepared to target and disassemble the *P. aeruginosa* biofilm by degrading extracellular DNA that stabilizes the biofilm matrix and released ciprofloxacin inside the biofilm to effectively eliminate *P. aeruginosa*, as reported by Torrents and coworkers [151].

**Figure 11:**
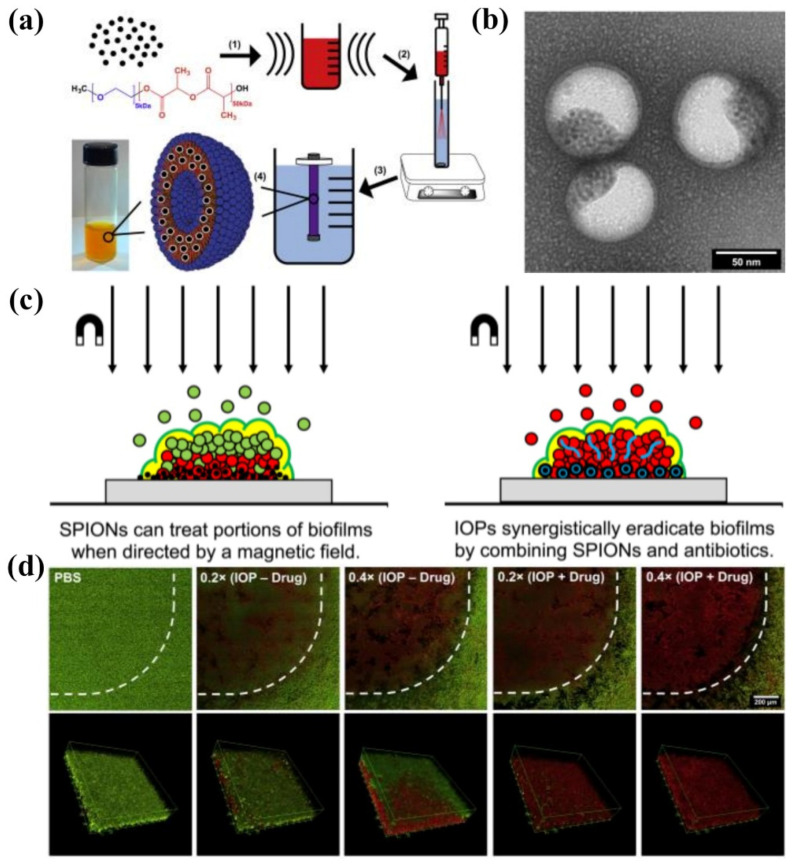
(**a**) Synthesis of IOPs loaded with SPION and methicillin. (1) 5 nm monodisperse hydrophobic SPIONs are combined with mPEG-PDLLA co-polymer in organic solvent and ultrasonicated to create a uniform suspension. (2) This organic phase is injected through an atomizer into an actively stirring aqueous phase containing PBS and methicillin. (3) The mixture is dialyzed against pure PBS to remove the organic solvent and unencapsulated drug to yield (4) highly stable polymersome solution. (**b**) TEM image of SPIONs loaded polymersomes. (**c**) Magnetic field induced treatment of biofilm using SPIONs and/or antimicrobials. (**d**) Confocal microscopy images of LIVE/DEAD staining of *S. epidermidis* biofilms treated with IOPs with an external magnetic field (reproduced with permission from Geilich et al. [152], Biomaterials; published by Elsevier, 2017).

Webster et al. [152] prepared bifunctional polymersomes with methicillin encapsulated in the hydrophilic cavity and superparamagnetic iron oxide nanoparticles (SPIONs) embedded in the membrane, as illustrated in Figure 11. The iron oxide-encapsulated polymersomes (IOPs) penetrated into the *S. epidermidis* biofilm with high efficiency, promoted by external magnetic field. Comparing with individual SPIONs, methicillin and SPION co-encapsulated polymersomes showed enhanced penetration capability up to 20 μm due to the improved relaxivity and magneticity (Figure 11c). Thus, methicillin could be released into the deep end of the biofilm, resulting in the effective eradication of pathogens. The confocal microscopy images and the 3D reconstructions of z-stacks of the bacterial biofilm revealed the capability of IOPs to eradicate biofilms with and without methicillin, as shown in Figure 11d. When there was no methicillin, only bacteria in the bottom layer of the biofilm were killed. On the contrary, all bacteria throughout the biofilm were eliminated by the methicillin loaded IOPs. These organic/inorganic hybrid nanocarriers showed great promise as new weapons for eradicating persistent biofilm or drug-resistant bacteria.

**Figure 12:**
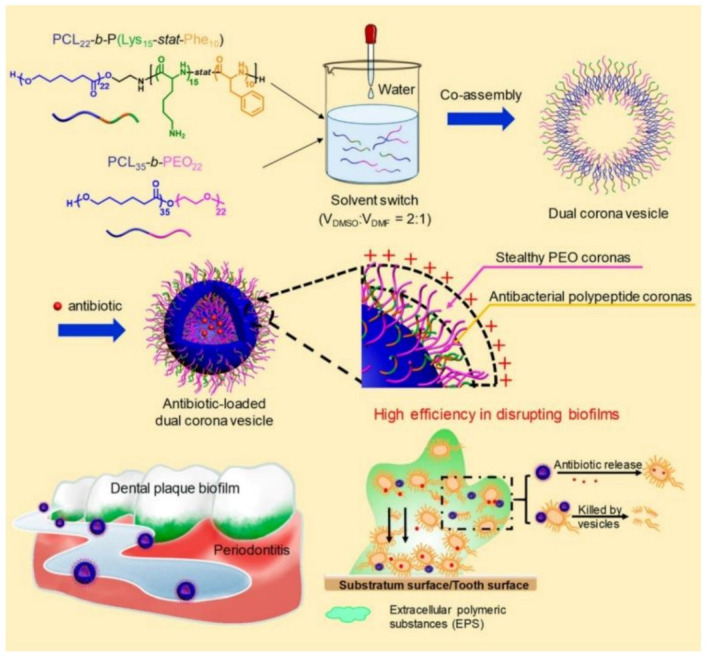
Schematic model showing the treatment of periodontitis by efficiently disrupting biofilms via antimicrobials -loaded multifunctional dual corona vesicles (reproduced with permission from Xi et al. [153], ACS Nano; published by American Chemical Society, 2019).

Recently, Du and coworkers [153] reported the treatment of periodontitis by efficiently disrupting biofilms using a dual corona antimicrobials-loaded polymer vesicle with stealthy poly(ethylene oxide) (PEO) corona to penetrate the biofilm and antibacterial polypeptide corona to provide intrinsic antimicrobial activity, as shown in Figure 12. The dual corona polymer vesicles were prepared by the co-assembly of two polymers PCL-*b*-poly(lysine-*stat*-phenylalanine) [PCL-*b*-P(Lys-*stat*-Phe)] and PEO-*b*-PCL with the same hydrophobic biodegradable PCL segment and different hydrophilic chains. Ciprofloxacin could be efficiently encapsulated in the cavity of the vesicles. Due to the protein-repelling ability of PEO, dual corona polymer vesicles penetrated the EPS of the biofilms with high efficiency, while the positive charged P(Lys-*stat*-Phe) allowed the vesicle to target and kill bacteria via electrostatic interaction. In addition, the encapsulated ciprofloxacin could be released as the polymer vesicle reached the deep end of the biofilm, resulting in a reduced dosage of the antimicrobials up to 50% to eradicate *E. coli* or *S. aureus* biofilms. In vivo experiment results demonstrated excellent performance of the dual corona vesicles in reducing dental plaque and alleviating inflammation using a rat periodontitis model.

Despite the strategy of delivering antibiotics to the deep end of biofilms by polymeric nanocarriers in order to reduce dosage and enhance antimicrobial activity, the efficient delivery of non-antibiotic antimicrobial agents including AMPs, AgNPs, photosensitizers and so forth for eliminating biofilms was also widely studied [154,155,156]. For instance, Haldar et al. [157] fabricated biodegradable polymer-coated AgNPs nanocomposite to eradicate biofilms, which reduced MRSA burden both on the catheter (>99.99% reduction) and in tissues surrounding the catheter (>99.999% reduction) in a mice model. Ji and coworkers [158] developed targeted photodynamic therapy strategies by using a supramolecular delivery system for the treatment of biofilms. The photosensitizer Chlorin e6 was grafted onto *α*-cyclodextrin, and the targeting group AMP Magainin I was covalently bound with PEG. Taking advantage of supramolecular recognition between *α*-cyclodextrin and PEG, targeting supramolecular micelles loaded with Chlorin e6 were formed, which exhibited excellent bacterial targeting effects and enhanced biofilm eradication ability against *P. aeruginosa* biofilm and MRSA biofilm. These results proved the versatility and great potential of polymeric nanomaterial-based antimicrobial agent delivery systems for eradicating biofilms.

### 3.3. Wound Healing

Wound infections induced by pathogens have become one of the main problems in wound care management systems, which impede the healing process and may result in life threatening complications. One of the approaches for treating wound infection is the use of wound dressings with antibacterial agents possessing broad-spectrum antimicrobial activity [159]. Typically, the moisture environment provided by the dressing has been shown to promote ulcer healing and to reduce pain experienced by patients [160]. Moreover, there are other requirements for wound dressings such as separating the wound with external environments and providing good breathability to promote wound healing. Polymeric nanomaterial-based delivery systems have shown considerable potentials in wound healing, especially polymer nanofibers and hydrogels [99,161]. For example, Lakshminarayanan et al. [162] prepared polydopamine crosslinked polyhydroxy antimicrobials loaded gelatin nanofiber mats for advanced wound dressings with long-term antimicrobial activity up to 20 days. The morphology of the nanofiber mats was retained for 1 month in an aqueous environment and showed comparable wound closure compared to commercially available silver-based dressings. Cai and coworkers [163] prepared composite hydrogels embedded with copper nanoparticles that could effectively convert NIR laser irradiation energy into localized heat for photothermal therapy. The synergistic effect of photothermal performance and rapid release of copper ions upon laser irradiation were responsible for excellent antimicrobial activity, reduced inflammatory response and promoted angiogenesis ability.

Antimicrobial agents including AMPs [49], antibiotics [164], AgNPs [165], metal oxide such as ZnO [166], photothermal sensitizers including porphyrin [167] and heavy metal ions [163] are usually used to improve the antimicrobial activity of polymeric wound dressings by covalent linkage, physical interaction or encapsulation. For example, Liu et al. [168] decorated chloramine on the surface of chitosan films by electrostatic interaction to heal MRSA infected wounds. Zhou and coworkers [167] prepared porphyrin containing alternating copolymer vesicles for the disinfection of drug-resistant bacteria infected wounds via photothermal effect. Fahimirad et al. [169] loaded recombinant LL37 AMP into chitosan nanoparticles for the elimination of MRSA infection during wound healing process with ultrahigh encapsulation efficiency of 78.52% and improved the activity and stability of LL37 AMP under thermal, salts and acidic pH treatments. Guo et al. [170] prepared injectable antimicrobial conductive quaternized chitosan hydrogels by loading graphene oxide via covalent bond for drug resistant bacterial disinfection and infectious wound healing, and the hybrid hydrogels showed excellent performance in the treatment of MRSA infected full-thickness defect mouse model.

**Figure 13:**
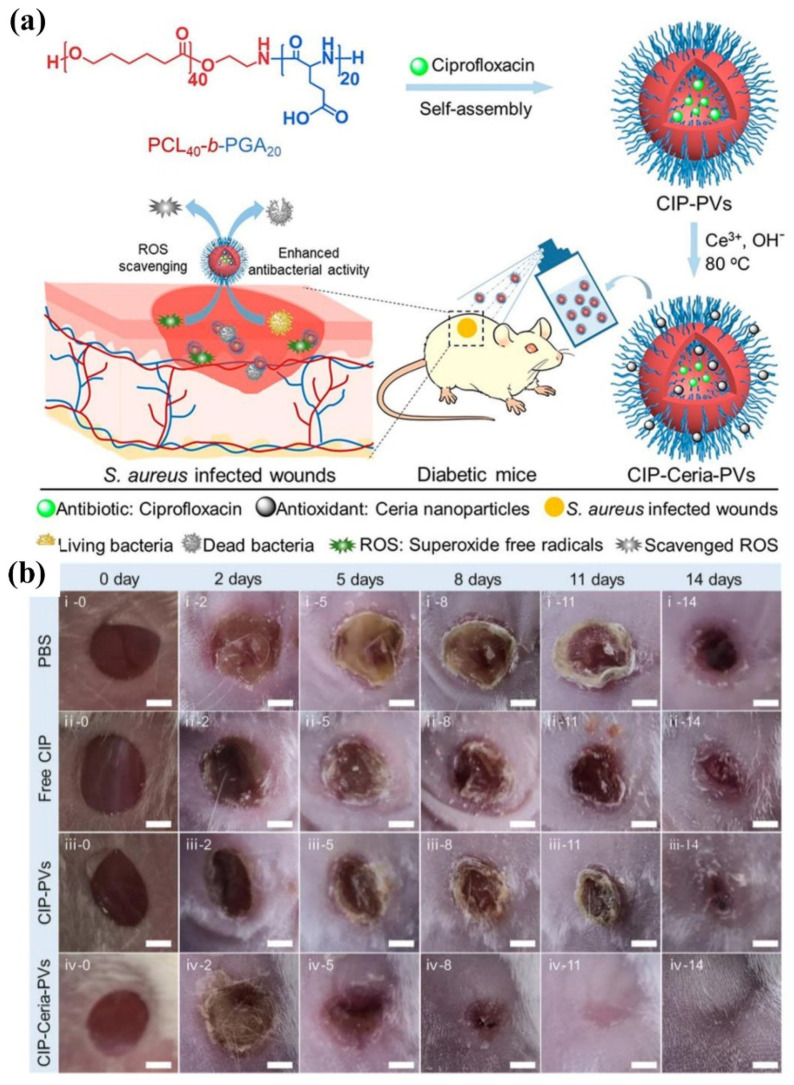
(**a**) Illustration of the preparation of CIP-Ceria-PVs and the combined antioxidant-antimicrobials treatment of infected diabetic wounds. (**b**) Digital images of infected diabetic wounds at different time intervals under treatment. Scale bar: 2 cm (reproduced with permission from Wang et al. [173], ACS Nano; published by American Chemical Society, 2021).

Very recently, polymer vesicles loaded with antimicrobials have been explored as dressings in promoting wound healing by spraying onto wounds [167,171,172,173]. Du and coworkers [173] reported bifunctional polymer vesicles loaded with antimicrobials and antioxidant for healing infected diabetic wounds, as presented in Figure 13. As one of the chronically infected wounds, the diabetic wounds are difficult to heal due to high ROS concentration and recurrent infections, resulting in the occurrence of diabetic ulcers and chronic diabetic complications with very high mortality rate. Therefore, scavenging ROS is very important in the treatment of diabetic wounds. In this study, well-dispersed ceria nanoparticles were deposited on the membrane of ciprofloxacin-loaded polymer vesicles (CIP-Ceria-PVs). The CIP-Ceria-PVs could inhibit peroxide free radicals up to 50% at extremely low cerium concentrations of 1.25 μg mL^−1^, protecting normal L02 cells from the damage of peroxide free radicals. Moreover, CIP-Ceria-PVs exhibited enhanced antimicrobial activity compared with free ciprofloxacin due to scavenging ROS. In vivo studies in Figure 13b demonstrated the excellent wound healing capability of CIP-Ceria-PVs, and the diabetic wound was completely healed within 14 days. At the same time, they developed a H_2_S delivery polymer vesicle, which was capable of long-term H_2_S generation to promote the proliferation, migration of epidermal and endothelial cells and angiogenesis, accelerating the complete healing of diabetic wounds [172].

### 3.4. Tissue Engineering

The regeneration of adult tissue following an injury or degeneration is quite a limited process. Usually, the injury site is vulnerable to bacterial infections, which causes complications and delay of the regeneration of tissues [174]. Therefore, the prerequisite of tissue regeneration is to eliminate localized bacterial infections, followed by the delivery of bioactive molecules such as growth factor to the defected tissues. Antimicrobial polymer coatings on the surface of implants can provide appropriate biointerfaces to promote the regeneration of tissues. For instance, ZnO nanoparticles embedded PLA was dip coated on magnesium alloy, which helped to control the degradation and increase antibacterial activity [175]. Suteewong et al. [176] deposited polymethylmethacrylate (PMMA)/chitosan-silver hybrid nanoparticles on rubber substrate, which exhibited enhanced antibacterial activity toward *E. coli* and *S. aureus* and reduced cytotoxicity to L-929 fibroblast cells, demonstrating the potential of this hybrid nanoparticle coating at soft substrates. In addition, antimicrobial agents loaded with polymer nanomaterials can be used as bioadhesives to repair damaged soft tissues. Gu and coworkers [177] developed fast and high strength bioadhesives based on polysaccharides and peptide dendrimers with inherent hemostatic ability and antibacterial properties. Moreover, the bioadhesive showed a remarkable 5-fold increase in adhesion strength comparing with commercial bioadhesive Coseal.

Biocompatible polymeric nanoparticles have been investigated as delivery vehicles for various tissue engineering applications [178]. For example, Du and coworkers [179] prepared antibacterial peptide-mimetic alternating copolymers (PMACs) vesicles loaded with growth factor for bone regeneration. They designed a series of PMACs with different repeating units, and the PMAC with a repeating unit of 14 exhibited the best antibacterial activity against both *E. coli* and *S. aureus* with ultralow MICs of 8.0 μg mL^−1^. After self-assembling into vesicles in pure water, the antimicrobial activity of the vesicles was well-preserved. Growth factor could be encapsulated in antimicrobial vesicles and released during the long-term antibacterial process to promote the regeneration of bone with a 20 mm defect model in rabbits. Micro-CT, bone mineral content and BMD were used to evaluate the repair of bone defects with scaffolds at 4 weeks and 6 weeks after implantation. After 6 weeks, the defect in the rabbit bone was completely repaired, demonstrating the excellent bone repair capability of antimicrobial growth factor-loaded vesicles.

### 3.5. Anticancer

The anticancer application of antimicrobial agents is an attracting field since cancers are often accompanied by inflammation, and the drug resistance of cancer cells is becoming increasingly concerning [180]. Theoretically, antimicrobial agents that kill bacteria via non-selective behaviors such as damage of the cell membrane [181], elevating temperature [182] and induced degeneration of proteins and genetic materials [183] can also kill cancer cells. For instance, Shim et al. [183] prepared AgNPs loaded chitosan-alginate composite, exhibiting broad-spectrum antimicrobial activity and high toxicity toward breast cancer cell line MDA-MB-231; Jothivenkatachalam and coworkers [184] fabricated chitosan-copper nanocomposite for the inhibition of various microorganisms and A549 cancer cells by photocatalytic effect. In addition, AMPs with specific sequences and proper positive charge densities have shown anticancer and antiviral activities, such as cecropin A and B, magainins, melittin, defensins, lactoferricin and so forth, as summarized by Hoskin’s and Franco’s group, respectively [181,185]. However, the AMPs are vulnerable to enzymes and can easily cause immune responses; thus, the delivery system is critical for in vivo applications of AMPs. Hazekawa et al. [186] conjugated antimicrobial human peptide, LL-37 peptide fragment analog, with a PLGA copolymer. The formed micellar system significantly improved the permeability of the peptide to cancer cells, and the proliferation, migration and invasion in various cancer cell lines were effectively exhibited. The intracellular delivery of peptides by polymer carriers in oncology applications has been summarized by Pun et al. very recently [187].

**Figure 14:**
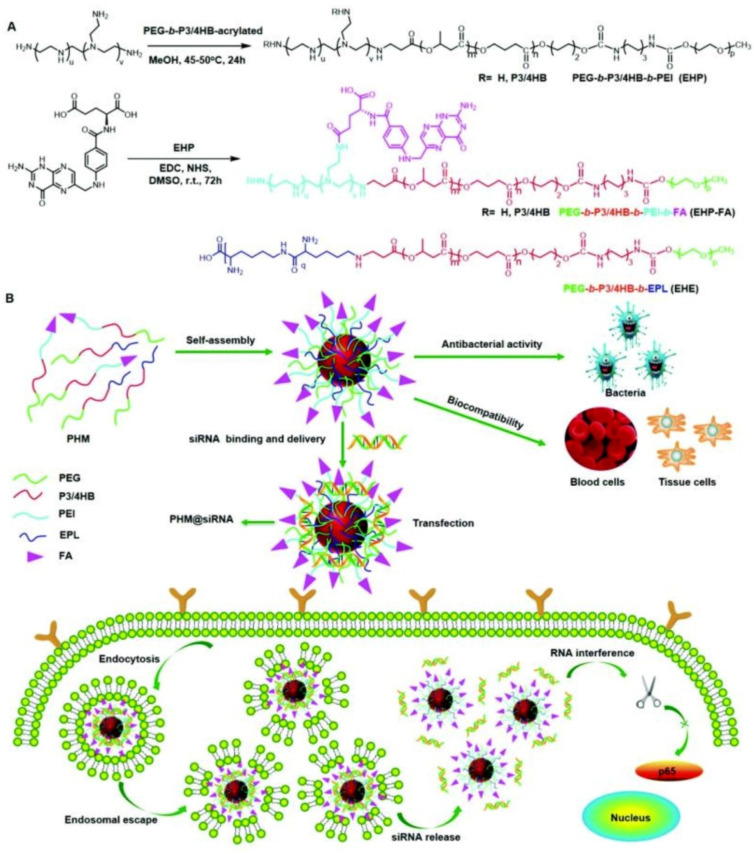
Scheme showing the synthesis and potential application of the PHM copolymer in siRNA delivery. (**A**) EHP and EHP-FA were synthesized by Michael addition and esterification reaction, respectively; PHM micelles were prepared by mixing EHP-FA and EHE copolymer; (**B**) schematic illustration of the application of PHM in siRNA delivery (reproduced with permission from Zhou et al. [192], Nanoscale; published by Royal Society of Chemistry, 2018).

Another strategy for eliminating cancer cells using antimicrobial delivery systems is the co-delivery of antimicrobial and anticancer agents simultaneously or loading anticancer drugs with antimicrobial carriers [188,189]. For instance, Du and coworkers [190] proposed the concept of “armed” carrier to co-deliver anticancer and antiepileptic drugs with antibacterial polypeptide-grafted chitosan-based nanocapsules. Mahkam et al. [191] designed pH-responsive antibacterial clay/polymer nanocomposite as a carrier to deliver anticancer drug methotrexate and antibacterial agent ciprofloxacin with an ultrahigh efficiency of >90%, which showed enhanced antimicrobial and anticancer activity compared with free methotrexate and ciprofloxacin, demonstrating the potential of antibacterial nanocarriers in cancer therapy. Lei and coworkers [192] developed a class of multifunctional polymeric hybrid micelles (PHM) with high antibacterial activity for the efficient delivery of siRNA to cancer cells, as illustrated in Figure 14. The PHM was prepared by the co-assembly of EHP-FA and EHE, for which their structures were presented in Figure 14A. Due to the existence of positively charged poly(ethylene imine) (PEI) and poly-ε-l-lysine (EPL), the PHM showed high antibacterial activity against *S. aureus* in vitro and in vivo. On the contrary, PHM exhibited good hemocompatibility and lower cytotoxicity toward A549, HeLa, HepG2 and C2C12 cells benefiting from the shield effect of PEG. siRNA could be complexed onto PHM by electrostatic interaction, and PHM with folic acid decorated on the surface could effectively target FA receptor overexpressed HeLa cells and other low-expressed cancer cells, resulting in the targeted delivery of siRNA. In vitro experiments revealed that the PHM showed a high p65 gene silencing efficiency above 90% in various cancer cells, which is significantly higher than EHP-FA and EHE, demonstrating the potential of PHM as a safe and effective siRNA vector with high antibacterial activity for multifunctional gene therapy.

## 4. Conclusions and Future Perspectives

In summary, the recent progress of efficient loading, delivery and controlled release of antimicrobial agents in vivo or in vitro by polymeric nanomaterial-based delivery systems have been concluded. A large diversity of antimicrobial agents including antibiotics, AMPs, AgNPs, metal nanoparticles, metal oxides, gases, photosensitizers and so forth could be loaded and delivered by polymeric nanomaterials either by physical interactions or covalent bonding while maintaining the intrinsic antimicrobial activity of these antimicrobial agents. In order to fit the physiochemical properties of different kinds of antimicrobial agents to construct highly efficient delivery systems with superiorities such as high loading content and efficiency, good stability and on-demand release, polymeric nanomaterials with different chemical compositions and nanostructures including micelles, vesicles, dendrimers, nanofibers and nanogels etc. are developed. Benefiting from the versatility of polymeric nanomaterials, the antimicrobial agent delivery systems have shown significant potentials in a wide variety of biomedical applications, such as combating MDR bacteria, anti-biofilm, wound healing, tissue engineering and anticancer. Despite the rapid development of this field, the in vivo and intracellular delivery of antimicrobial agents is still in its early stage, and there are numerous challenges that should be considered in the future, which may bring new opportunities in the biomedical applications of antimicrobial agent-based delivery systems.

Non-covalent interactions such as hydrogen bonding, π-π stacking and coordination should be introduced to enhance the interactions between antimicrobial agents and the polymeric nanocarriers to increase loading content and efficiency. The strong interactions could also prevent the leakage of cargoes before reaching the target and enhance the stability of the delivery system. Modulation of the properties of different kinds of antimicrobial agents and the structural features of carriers may maximize the efficiency of the loaded antimicrobial agents. Targeting the infected area and high selectivity toward bacteria rather than mammalian cells should always be considered, which is very important for the reduction in side effects and drug resistance. Moreover, external stimuli, especially non-invasive stimuli-triggered release of loaded antimicrobial agents (in other words, the switchable antimicrobial activity of the delivery system), are also helpful for the reduction in side effects and drug resistance. However, the spatial and temporal sensitivity of the stimuli-triggered response still needs to be improved to meet practical applications. Furthermore, the generations of antimicrobial active species such as ROS or change of the micro-circumstance including elevating temperature triggered by stimuli or chemicals secreted by bacteria are also effective methods for eliminating bacteria without the generation of drug resistance. Regardless of the generation of drug resistance, taking advantage of the synergistic effect of multiple antibacterial agents is an effective strategy for eradicating MDR bacteria. In addition, the combination of antibacteria and anticancer simultaneously will be of great significance in cancer therapy.

The biosafety of polymeric nanomaterial-based delivery systems has always been selectively ignored in previous studies. Although many biodegradable polymers have been used, the cytotoxicity and hemolytic activity of the polymeric carriers, especially those with positively charged surfaces, should be evaluated systematically. In addition, the word “biocompatibility” is a comprehensive evaluation of in vivo delivery systems. If we claim that the carrier is biocompatible, numerous parameters should be evaluated more than cytotoxicity and hemolytic activity. The in vivo delivery of antimicrobial agents has been reported in many studies. However, very few investigated the stability of the delivery system in physiological conditions and the interactions between the carriers and proteins, salts, glucose, fatty acids, antigens and so forth. Moreover, the immune response of the delivery systems is also hardly investigated. Considering the complexity of the physiological condition, it is necessary to reveal the stability and true circulation behavior of the delivery systems in vivo and not only borrowing the results of in vitro experiments. Furthermore, the full life-cycle assessment of polymeric carriers should be conducted to explore blood circulation behavior, biodistribution, metabolism and organic accumulations, etc., which will be very valuable for the instructive design of polymer carriers to promote the clinical applications of polymeric nanomaterials-based antimicrobial delivery systems.
